# Rabies Cluster Among Steers on a Dairy Farm — Minnesota, 2024

**DOI:** 10.15585/mmwr.mm7440a3

**Published:** 2025-12-11

**Authors:** Carrie Klumb, Malia Ireland, Bonnie Miller, Erik Jopp, Betsy Lempelius, Albert Rovira, Hemant Naikare, Carly Bauer, Katie Harry, Scott A. Cunningham, Gongping Liu, Thomas F. Czeck, Ryan Wallace, Brian Hoefs, Stacy Holzbauer

**Affiliations:** ^1^Minnesota Department of Health; ^2^Minnesota Board of Animal Health; ^3^University of Minnesota Veterinary Diagnostic Laboratory, St. Paul, Minnesota; ^4^Freeport Veterinary Service, Freeport, Minnesota; ^5^Division of High Consequence Pathogens and Pathology, National Center for Emerging and Zoonotic Infectious Diseases, CDC; ^6^Career Epidemiology Field Officer Program, CDC.

**Summary**

**What is already known about this topic?**

Rabies is a viral disease that infects mammals and has an almost 100% fatality rate if postexposure prophylaxis is not administered before symptom onset. Outbreaks among cattle are rare but have been reported.

**What is added by this report?**

During a 4-week period in May 2024, five of 35 steers on a Minnesota dairy farm developed neurologic signs consistent with rabies. The cluster likely represents a point-source exposure to a rabid skunk; however, steer-to-steer transmission could not be ruled out.

**What are the implications for public health practice?**

Rabies in cattle can expose humans to the disease and result in economic loss for farmers. Preventive vaccination of cattle should be considered to prevent human exposure in areas where rabies is prevalent and the value of the livestock is high.

## Abstract

Rabies clusters in domestic livestock are rare but can result in human exposure and economic loss for farmers. During a 4-week period in May 2024, five of 35 steers on a Minnesota dairy farm developed neurologic signs consistent with rabies. Three clinically ill steers were euthanized, and brain specimens were submitted for rabies testing. Direct fluorescent antibody testing and whole genome sequencing confirmed rabies virus (North Central Skunk variant) in all three steers. After identification of the first two rabid steers, the remaining animals were quarantined for 120 days and vaccinated against rabies; three additional steers became ill during quarantine and were euthanized. The Minnesota Department of Health and Minnesota Board of Animal Health investigated human and animal exposures through interviews and site visits. Five persons were recommended to receive rabies postexposure prophylaxis because of known or potential exposures. The outbreak likely resulted from a single rabid skunk biting multiple cattle housed in a small pen, although steer-to-steer transmission cannot be ruled out. In addition to the loss of livestock, direct medical and veterinary costs associated with this outbreak totaled approximately $35,000. Preventive vaccination of cattle should be considered in areas with high activity of terrestrial rabies (i.e., rabies in land-based animals), presence of high-value livestock, and potential for human exposure.

## Introduction

Rabies is a vaccine-preventable, viral, zoonotic disease that affects the central nervous system of mammals (*1*). Rabies virus is typically transmitted through the bite of an infected mammal or mucous membrane exposure to the virus (*2*). In the United States, the wildlife reservoirs for rabies are bats (multiple species), raccoons, foxes, mongooses, and skunks (Rabies in the United States | CDC). Although rabies has an almost 100% fatality rate, prompt administration of rabies post exposure prophylaxis (PEP) after an exposure is highly effective at preventing disease (*2*,*3*). Rabies clusters in domestic livestock are rare but can result in human exposure and significant economic losses for farmers. Cattle are not routinely vaccinated against rabies.

## Investigation and Findings

### Identification of the First Rabies Cases

On May 11, 2024, a steer on a Minnesota dairy farm died a few days after the onset of neurologic signs including drooling, poor coordination, bellowing, and head thrashing; no necropsy was performed. On May 13, the farm owners sought veterinary care for a second steer exhibiting similar neurologic signs. The second steer was euthanized, and a sample of brain tissue was prepared at the University of Minnesota Veterinary Diagnostic Laboratory. Direct fluorescent antibody (DFA) testing of the brain tissue was performed at the Minnesota Department of Health (MDH) Public Health Laboratory and was positive for rabies on May 16. Whole genome sequencing (WGS) of the rabies virus was performed, which identified the North Central Skunk rabies virus variant. MDH and Minnesota Board of Animal Health (BAH) investigated to determine human and animal exposures. These activities were reviewed by CDC, deemed not research, and were conducted consistent with applicable federal law and CDC policy.*

### Investigation and Management of Animals on the Farm

A BAH district veterinarian visited the farm premises to conduct an investigation, which included interviewing the farm owners, verifying animal vaccination histories, assessing potential rabies exposures among the animals, and establishing any necessary animal quarantines. During this investigation, the farm owners reported smelling a skunk several weeks before the onset of the first steer’s illness, although no bite to the steer was identified. None of the animals in the herd had received rabies vaccination. To decrease the risk for rabies in additional steers in the herd and human exposure to rabid animals, the herd veterinarian initiated a 2-dose regimen of postexposure rabies vaccination for the remaining 33 steers, with the first dose administered on May 18 (Figure). Because two steers from the same pen had been infected, on May 24, BAH placed the 33 steers in a pen under a 45-day quarantine for rabies observation.

**FIGURE:**
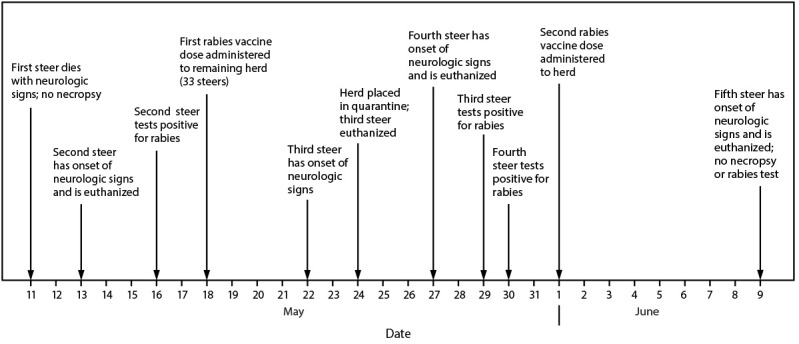
Rabies cases among steers on a dairy farm — Minnesota, May 11–June 9, 2024* * No additional cases were reported after June 9, 2024.

One dog, that was up-to-date on its rabies vaccine, lived on the farm and had access to the steer’s carcass before burial. The dog received a rabies vaccine booster dose on May 17, and BAH placed the dog on a 45-day observation period, which consisted of the owners watching the dog closely for signs of illness, limiting contact to those inside the household, and contacting BAH if clinical signs developed. The dog remained healthy. Per National Association of State Public Health Veterinarians guidance (*4*), 12 unvaccinated farm cats that did not have clinical signs of rabies but might have been exposed to the rabid steers were euthanized.

On May 24 and 27, two additional steers displayed neurologic signs similar to those observed in the first two steers and were euthanized; DFA testing of brain specimens was positive for rabies virus for both animals. Because additional cases were identified after the index case, BAH extended the quarantine of the herd to 120 days to ensure that any possible steer-to-steer transmission was detected.

On June 1, the 31 remaining steers received the scheduled second rabies vaccine dose. On June 9, a fifth steer developed signs similar to those observed among the previous four steers and died; no tissue from this animal was submitted for rabies testing. WGS of rabies virus isolated from the three tested animals (steers 2, 3, and 4) (Figure) indicated that the detected North Central Skunk rabies virus variant differed by at least single nucleotide polymorphisms. No additional steers died.

### Identification of Potentially Exposed Persons

MDH epidemiologists conducted interviews with six potentially exposed persons to determine whether rabies PEP was indicated. These included persons who might have had contact with saliva from or might have been bitten by a rabid steer and were not wearing appropriate personal protective equipment (i.e., gloves, eye protection, and face protection).

Five persons (one veterinarian, two farm owners, and two children) were determined to have had possible exposure to rabid steers. The veterinarian’s glove and skin were punctured during removal of an infected steer’s brain. The farm owners had extensive contact with all of the infected steers, including possible saliva contact. The children, aged <10 years, had unsupervised contact with the area where the steers were housed, and thus exposure could not be ruled out. All five persons received rabies PEP.^†^ The four family members received the full PEP series, and the veterinarian, who had received rabies vaccine before the exposure, received 2 booster doses.

## Public Health Response

On June 12, BAH published a rabies alert on its website providing details about the rabid steers, information on vaccinating pets and livestock, and recommendations for actions to take if an abnormally behaving skunk was identified. A press release summarizing the outbreak and providing educational resources and contact information to discuss potential rabies exposures was issued by MDH and published by select rural newspapers in Minnesota. The press release and subsequent articles informed residents of the increased number of rabid skunks and subsequent increased number of rabid cattle in Minnesota in 2024 (*5*). The press release and articles served as reminders to vaccinate pets, consider vaccinating livestock, and seek medical attention for any potential rabies exposures. Direct medical and veterinary costs for this outbreak included PEP for four persons ($32,000), booster vaccine doses for the veterinarian ($730), veterinary visits ($1,300), postexposure vaccination of steers ($889), rabies booster for the dog ($134), and specimen shipping and rabies testing ($218)^§^ (*6*).

## Discussion

An estimated 60,000 human exposures to rabies occur each year in the United States through exposure to wildlife and unvaccinated domestic animals (*7*). Striped skunks are the primary terrestrial (land-based) wildlife rabies reservoir in Minnesota. Forty-two percent of skunks submitted for rabies testing in Minnesota are confirmed positive (Animals tested for rabies | 2003–2024 | MDH); the rabies variant most commonly identified in infected domestic animals in Minnesota (including in cats, dogs, horses, and cattle) is the North Central Skunk rabies virus variant (MDH, unpublished data, 2015–2024).

Temporal clustering of rabid animals on a farm is unusual but has been reported (*4*). The dairy steers on this farm were housed in a small pen, making it possible for a rabid skunk to bite multiple animals. The incubation period for rabies in domestic animals can vary depending on the location and severity of the bite, and the rabies virus variant. Combined with WGS results that indicated closely related viruses, findings from this investigation suggest that multiple steers were infected by a single rabid skunk. However, a cluster of five rabid steers (three laboratory-confirmed cases and two with compatible clinical signs) during a 4-week period on one farm is highly unusual, and steer-to-steer transmission cannot be ruled out.

Rabid livestock can result in exposures to humans and economic loss to farmers; such losses are not typically reimbursed by farm business insurance and are also not part of the Department of Agriculture’s Livestock Indemnity Program, which provides benefits to livestock producers for deaths in excess of normal mortality. All associated veterinary costs, income loss from the five steers, and additional feed costs for the remaining herd were incurred by the owners. Although not routinely vaccinated against rabies, cattle are the livestock species most often infected (*8*). An economic analysis of rabies vaccination in cattle estimated U.S. losses associated with livestock rabies ranging from $9.7 million to $40.5 million during 2012–2021 (*8*). Preventive vaccination of cattle should be considered in areas where terrestrial rabies is prevalent, the value of the animals is high, and potential for human exposure exists (*8*).

### Limitations

The findings in this report are subject to at least two limitations. First, MDH was unable to procure brain tissues samples for DFA testing from the first and last dairy steers that exhibited abnormal neurologic signs and died. However, based on the similar signs and timing of illness and the fact that they were housed in the same pen as the three steers with confirmed rabies infection, rabies is likely also the cause of their deaths. Second, the source of rabies exposure could not be confirmed. While there was evidence of skunk activity on the farm, no skunk was observed on the property or submitted for rabies testing, and it was not possible to confirm that steer-to-steer transmission did not occur.

### Implications for Public Health Practice

Rabies is considered a fatal disease if PEP is not administered (Rabies | CDC; Minnesota's Rabies Facts | MNDOH). Temporal clustering of rabid animals on a farm is unusual but when it does occur it can result in potential human exposures and economic loss for the owners. In regions of the United States with a high level of rabies in terrestrial reservoirs, it might be worth evaluation of the benefits and costs of preventive vaccination of high-value animals to prevent human rabies and to potentially save animal owners from economic loss.

## Footnotes

* 45 C.F.R. part 46.102(l)(2), 21 C.F.R. part 56; 42 U.S.C. 241(d); 5 U.S.C. 552a; 44 U.S.C. 3501 et seq.

† Rabies PEP consists of 1 dose of human rabies immune globulin (HRIG) (20 IU per kg of body weight), followed by 4 rabies vaccine doses on days 0, 3, 7, and 14. HRIG is administered on the same day as the first rabies vaccine dose and is only indicated for persons who have not previously been vaccinated against rabies (Rabies post-exposure prophylaxis | CDC).

§ Cost information for rabies postexposure vaccination was obtained by interviewing the farm owners and the herd veterinarian. Veterinary costs to the farm owners were obtained from service bills provided by the herd veterinarian.
